# The Medical Journal of North Carolina

**Published:** 1858-09

**Authors:** 


### THE MEDICAL JOURNAL OF NORTH CAROLINA.

We have received the first number of a new Medical Journal, with the above title, published at Edenton, N. C., and edited by Edward Warren, M. D.

We are pleased with the appearance, as well as the contents of the Journal, and being the only one published in the State of North Carolina, it will surely be well supported. We cordially welcome it to our exchange list, and unite in the hope "that a protracted existence and exalted destiny awaits the Medical Journal of North Carolina."

It is a bi-monthly of "not less than one hundred pages," and furnished at $3 per annum.

